# NRF2 Signaling Negatively Regulates Phorbol-12-Myristate-13-Acetate (PMA)-Induced Differentiation of Human Monocytic U937 Cells into Pro-Inflammatory Macrophages

**DOI:** 10.1371/journal.pone.0134235

**Published:** 2015-07-29

**Authors:** Min-gu Song, In-geun Ryoo, Hye-young Choi, Bo-hyun Choi, Sang-Tae Kim, Tae-Hwe Heo, Joo Young Lee, Pil-Hoon Park, Mi-Kyoung Kwak

**Affiliations:** 1 College of Pharmacy, Yeungnam University, Gyeongsan, Gyeongsangbuk-do 712–749, Republic of Korea; 2 College of Pharmacy, The Catholic University of Korea, Bucheon, Gyeonggi-do 420–743, Republic of Korea; 3 Seoul National University Bundang Hospital, Sungnam, Gyeonggi-do 463–707, Republic of Korea; University of Padova, ITALY

## Abstract

Blood monocytes are recruited to injured tissue sites and differentiate into macrophages, which protect against pathogens and repair damaged tissues. Reactive oxygen species (ROS) are known to be an important contributor to monocytes’ differentiation and macrophages’ function. NF-E2-related factor 2 (NRF2), a transcription factor regulating cellular redox homeostasis, is known to be a critical modulator of inflammatory responses. We herein investigated the role of NRF2 in macrophage differentiation using the human monocytic U937 cell line and phorbol-12-myristate-13-acetate (PMA). In U937 cells with *NRF2* silencing, PMA-stimulated cell adherence was significantly facilitated when compared to control U937 cells. Both transcript and protein levels for pro-inflammatory cytokines, including interleukine-1β (IL-1β), IL-6, and tumor necrosis factor-α (TNFα) were highly elevated in PMA-stimulated *NRF2*-silenced U937 compared to the control. In addition, PMA-inducible secretion of monocyte chemotactic protein 1 (MCP-1) was significantly high in *NRF2*-silenced U937. As an underlying mechanism, we showed that *NRF2*-knockdown U937 retained high levels of cellular ROS and endoplasmic reticulum (ER) stress markers expression; and subsequently, PMA-stimulated levels of Ca^2+^ and PKCα were greater in *NRF2*-knockdown U937 cells, which caused enhanced nuclear accumulation of nuclear factor-ҡB (NFҡB) p50 and extracellular signal-regulated kinase (ERK)-1/2 phosphorylation. Whereas the treatment of *NRF2*-silenced U937 cells with pharmacological inhibitors of NFҡB or ERK1/2 largely blocked PMA-induced IL-1β and IL-6 expression, indicating that these pathways are associated with cell differentiation. Taken together, our results suggest that the NRF2 system functions to suppress PMA-stimulated U937 cell differentiation into pro-inflammatory macrophages and provide evidence that the ROS-PKCα-ERK-NFҡB axis is involved in PMA-facilitated differentiation of *NRF2*-silenced U937 cells.

## Introduction

Monocytes, which are differentiated from bone marrow hematopoietic stem cells, are heterogeneous circulating blood cells. Monocytes are recruited at the site of infiammation where they adhere to extracellular matrix molecules (ECM) or other immune cells via specific surface proteins, such as cluster of differentiation 44 (CD44) [1, 2]. Activated monocytes undergo differentiation programs to rapidly mature into macrophages. Stimulation by invading microbes and T-cell derived cytokines leads to the classical macrophage activation and production of cytokines, such as interleukin-1β (IL-1β), IL-6, IL-13, tumor necrosis factor-α (TNFα), and nitric oxide (NO) [1, 3]. During this process, macrophages acquire heterogeneous phenotypic characteristics depending on their environmental conditions to exhibit functional diversity [4]. There is considerable evidence that reactive oxygen species (ROS), which are produced from NADPH oxidase during macrophage activation, assist in eliminating pathogens and are an important component of signaling network toward inflammatory responses [3, 5].

Due to the limited availability of primary tissue macrophages, monocytic cell lines such as U937 and THP-1 are often used as a model of macrophages. U937 cells can undergo monocyte/macrophage differentiation after several types of stimulations. As a physiological stimulation, interferon-γ (IFN-γ) treatment induces differentiation of U937 cells into macrophage [6]. As an exogenous chemical, phorbol esters such as phorbol-12-myristate-13-acetate (PMA) are well-studied differentiation-inducing chemicals [7–9]. Since PMA is an analog of diacyl glycerol (DAG), which is a strong activator of protein kinase C (PKC), PMA treatment stimulates PKC signaling cascade in U937 to alter the expression of a wide range of genes via multiple transcription factors, including nuclear factor-κB (NFκB) and activator protein-1 (AP-1) [10–13]. In particular, NFκB is a critical factor for the expression of pro-inflammatory cytokines [14]. Transcription activity of NFκB is primarily regulated by cytosolic inhibitory protein IκB, which is a substrate of protein kinase IκB kinase (IKK) [15]. In addition, various signaling pathways involve NFκB activation: Mitogen-activated protein kinases (MAPKs) such as extracellular signal-regulated kinase (ERK) phosphorylate NFκB and activate its signaling pathway [16, 17]. PMA-stimulated PKC can enhance NFκB signaling directly as well as through MAPK-mediated pathway. Overexpression of PKCα and PKCε induces NFκB activation in T cells. PKCζ mediates phosphorylation of p65 to facilitate CREB binding protein (CBP) recruitment and NFκB activation [18, 19].

Endoplasmic reticulum (ER) stress can be caused by the accumulation of unfolded/misfolded proteins within the ER. This state activates the unfolded protein response (UPR) pathways involving three signaling molecules located in the ER membrane: protein kinase R-like endoplasmic reticulum kinase (PERK), IRE1α, and activating transcription factor 6 (ATF6) [20, 21]. Activation of these ER stress-sensing molecules recovers ER homeostasis by alleviating protein synthesis speed, increasing chaperone molecules, and enhancing protein degradation. In addition, it is notable that ER stress initiate tissue inflammation. The ER stress-induced UPR signaling activates the NFκB and MAPKs pathways to induce cytokine expression [21].

The transcription factor NF-E2-related factor 2 (NRF2) governs the expression of a myriad of genes encoding antioxidant proteins and detoxifying enzymes, including γ-glutamate cystein ligase (GCL), heme oxygenase-1 (*HO-1*), NAD(P)H quinone oxidoreductase (*NQO-1*), and aldo-keto reductases (AKRs) [22, 23]. NRF2 regulation is primarily mediated by Kelch-like ECH-associated protein 1 (KEAP1) [24]. Without stimulators, the KEAP1-bound NRF2 protein is subjected to proteasomal degradation through Cullin 3 (CUL3)-based E3 ligase-mediated ubiquitination. When cells are exposed to oxidizing conditions or NRF2 activators, cysteine residues of the KEAP1 protein are modified, and therefore, NRF2 escapes from KEAP1-mediated degradation pathway, resulting in the transactivation of genes bearing the antioxidant response element (ARE) in their promoters. In addition, multiple protein kinases, such as PKC and MAPKs, have been reported to contribute to NRF2 activation by enhancing NRF2 liberation from KEAP1 or NRF2 nuclear shuttling [23]. Due to antioxidative and cytoprotective functions of NRF2 target genes, NRF2 is widely accepted as a multi-organ protector [25, 26]. In particular, the NRF2 system is known to suppress inflammatory responses in animal macrophages [27, 28].

Based on reports of the role of NRF2 on macrophage activity, we questioned whether NRF2 affects monocyte’s differentiation into macrophages. Since ROS are critical factors involved in determining monocytes differentiation and macrophage activation, there is a possibility that NRF2 can control this process. Therefore, we investigated the involvement of NRF2 in PMA-induced monocyte’s differentiation using *NRF2*-silenced U937 cells as a model of macrophage-like cells, and further suggested signaling pathways associated with differentiation.

## Materials and Methods

### Materials

Antibodies for NFκB p50 (3035), IκB (9242), PKCα (2056), phosphorylated initiation factor 2α (p-EIF2α, 9721), phosphorylated PERK (3179), ERK1/2 (9102), p-ERK1/2 (9101), and glyceraldehyde 3-phosphate dehydrogenase (GAPDH, 2118) were obtained from Cell Signaling Technology (Beverly, MA, USA). The β-tubulin antibody (sc-9104) was obtained from Santa Cruz Biotechnology (Santa Cruz, CA, USA). PD98059 and BAY11-7082 were purchased from Calbiochem (Billerica, MA, USA). Fluo-4 acetoxymethyl (AM), 2',7'-dichlorodihydrofluorescein diacetate (H_2_DCFDA), Alex 488, Hoechst 33342, 4',6-diamidino-2-phenylindole (DAPI), and Nucblue were purchased from Life Technologies (Carlsbad, CA, USA). The lentiviral system containing a pre-designed human *KEAP1* shRNA was obtained from Sigma Aldrich (Saint Louis, MO, USA). Other reagents, including PMA and propidium iodide (PI), were purchased from Sigma-Aldrich.

### Cell culture and PMA-induced differentiation

Human monocytic cell line U937 (CRL-1593.2; American Type Culture Collection, Manassas, VA, USA) was maintained in RPMI-1640 medium with 10% defined fetal bovine serum (Hyclone, Logan, UT, USA) and penicillin/streptomycin (Welgene Inc., Daegu, South Korea). Cells were maintained at 37°C in a humidified 5% CO_2_ atmosphere. For differentiation, U937 cells were grown overnight on a 6-well plate at a density of 8 × 10^5^ cells per well. Cells were then incubated with vehicle (ethanol) or PMA (1, 2.5, or 10 ng/mL) for 24 h and washed with PBS to remove non-adherent cells. Adherent cells were photographed using a microscope (Carl Zeiss, Jena, German), and the number of adherent cells were counted.

### Establishment of *NRF2*-silenced U937 cells

U937 cells were transduced with lentiviral particles containing either nonspecific scRNA (pLKO.1-scRNA) or *NRF2* shRNA (pLKO.1-*NRF2* shRNA) expression plasmids, and stable cell lines were established as previously described [29].

### Total RNA extraction and real-time RT-PCR

Total RNAs were isolated from cells by using TRIzol reagent (Invitrogen, Carlsbad, CA, USA). For cDNA synthesis, RT reaction was performed by incubating 200 ng of total RNA with a reaction mixture containing 0.5 μg/μL oligo dT_12–18_ and GoScript RT (Promega, Madison, WI, USA). Real-time reverse transcriptase (RT)-polymerase chain reaction (PCR) analysis was performed using a Roche LightCycler (Mannheim, Germany) with the Takara SYBR Premix ExTaq system (Otsu, Japan) as described previously [30]. The relative expression level of each gene was normalized using the housekeeping gene hypoxanthine-guanine phosphoribosyltransferase (HPRT) or GAPDH. Primer sequences for *NRF2*, *NQO1*, the modulatory subunit of GCL (*GCLM*), *AKR1c1*, and *HPRT* are described in our previous study [29]. Primers for the human X-binding protein-1 (XBP-1) are as follows: 5′-CCTGGTTGCTGAAGAGGAGG-3′ and 5′-CCATGGGGAGATGTTCTGGAG-3′. PCR amplification for *XBP1* gene was carried out with a thermal cycler (Bio-Rad, Hercules, CA, USA) and amplification conditions were 40 cycles of 40 s at 95°C, 30 s at 56°C and 30 s at 72°C. PCR products were resolved on 3% agarose gels and the images were captured by using a Gel Doc EZ Imager (Bio-Rad, Hercules, CA, USA). All primers were synthesized by Bioneer (Daejeon, South Korea).

### Western blot

Cells were lysed with radioimmunoprecipitation assay (RIPA) buffer (1 M pH 7.4 Tris, 2 M NaCl, 1 M EDTA, and 10% NP40) and protein concentration was measured using a BCA protein assay kit (Thermo Scientific, Waltham, MA, USA). Protein samples were electrophoresed on 6–10% SDS-polyacrylamide gels and transferred onto nitrocellulose membranes (Whatman GmbH, Dassel, Germany) as described previously [31]. The membrane was blocked with 5% skim milk for 1 h, and incubated with the primary antibody overnight. Following secondary antibody incubation, chemiluminescent signal was detected using the Supersignal West Pico chemiluminescent substrate (Thermo Scientific) with LAS-4000 mini imager (Fujifilm, Tokyo, Japan).

### Preparation of nuclear extracts

Crude nuclear fractions were prepared by lysing cells with the homogenization buffer (2 M sucrose, 1 M HEPES, 2 M MgCl_2_, 2 M KCl, 30% glycerol, 0.5 M EDTA, 1 M DTT, 0.5% NP40, and a protease inhibitor cocktail) and centrifugation at 12,000 g for 15 min.

### Immunocytochemical analysis

U937 cells were cultured in 35 mm dish with coverslip at a density of 2 × 10^3^ cells/mL. The next day, the cells were washed with cold PBS and fixed with ice-cold methanol or 4% formaldehyde for 10 min. After permeabilization, cells were incubated with anti-p50 or anti-PKCα antibodies at 4°C for 1 h. The cells were then incubated with Alexa Flou 488 (Invitrogen) and conjugated with secondary antibodies (1:200) for 90 min at room temperature. Hoechst 33342 was used for nucleus staining. Fluorescent images were acquired using an LSM 710 confocal microscope (Carl Zeiss, Jena, Germany) and ZEN 2011 software (Carl Zeiss) as described previously [32].

### Measurement of intracellular ROS

Cell-permeable fluorogenic probe carboxy-H_2_DCFDA was used to determine ROS cellular levels [33]. U937 cells in 35 mm dish were incubated with 30 μM of carboxy-H_2_DCFDA for 30 min at 37°C. Fluorescent images were obtained using an appropriate filter (488/524 nm) with LSM 710 confocal microscope (Carl Zeiss, Jena, Germany) and intensities were quantified using the ZEN2011 software (Carl Zeiss). For nuclei staining, Hoechst 33342 was used.

### Determination of intracellular Ca^2+^

U937 cells in RPMI 1640 were allowed to settle on 35 mm dish-attached coverslips for 12 h. The cells were incubated with 2 μM Fluo-4 AM without FBS for 30 min at 37°C, and excess Fluo-4 AM was washed off. Right after PMA addition, fluorescent images were determined using the confocal microscope (LSM 710). The mean fluorescence intensity of six to eight cells in the visual field was measured using the ZEN2011 software.

### Multiplex cytokine assay

The scRNA-expressing and NRF2 shRNA-expressing stable U937 cell lines were incubated with PMA for 24 h, and culture media were collected. Levels of IL-1β, IL-6, TNFα, and monocyte chemotactic protein 1 (MCP-1) within the collected media were analyzed with the Bio-Plex assay system (Bio-Rad) according to the manufacturer’s protocol.

### Statistical analysis

Two-way ANOVA and un-paired Student’s t-test were used to determine statistical significances (GraphPad Prism software, La Jolla, CA, USA). P < 0.05 was considered statistically significant.

## Results

### PMA-induced adherence is enhanced in *NRF2*-silenced U937 cells

**Fig 1:**
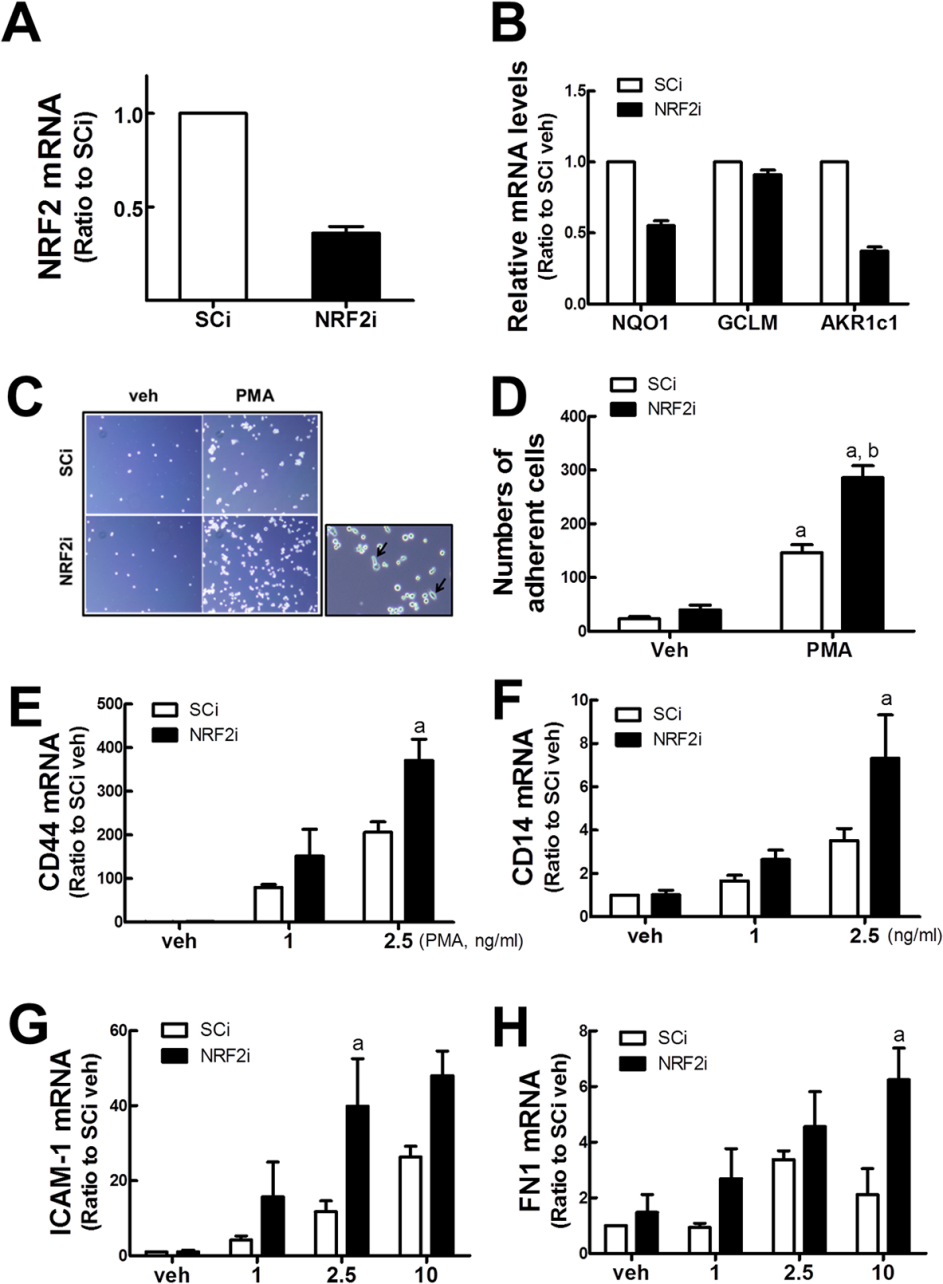
PMA-stimulated adherence of NRF2i U937 cells. (A) Transcript levels of NRF2 in SCi and NRF2i U937 cells were measured by semi-quantitative real-time RT-PCR. (B) Transcript levels for NRF2-target genes, *NQO1*, *GCLM*, and *AKR1c1*, were determined by semi-quantitative real-time RT-PCR analysis. The expression levels of each gene were normalized with respect to the housekeeping gene HPRT. Data represent the means ± SD of 3–4 experiments. (C-D) SCi and NRF2i cells were incubated with vehicle (veh, ethanol) or PMA (10 ng/mL) for 24 h, and adherent cells were photographed following PBS washing (C). Arrows indicate cells with a macrophage-like morphology. Numbers of adherent cells were counted in microscopic images (D). Data represent the means ± SD of 3 microscopic areas. ^a^P < 0.05 compared with vehicle control. ^b^P < 0.05 compared with PMA-treated SCi cells. (E-H) Transcript levels of adhesion molecules in PMA-treated NRF2i U937 cells. SCi and NRF2i U937 cells were incubated with vehicle (ethanol) or PMA (1, 2.5, and 10 ng/mL) for 24 h, and transcript levels for adhesion molecules CD44 (E), CD14 (F), ICAM-1 (G), and extracellular matrix FN1 (H) were assessed by real-time RT-PCR analysis. Expression levels of each gene were normalized with respect to the housekeeping gene HPRT or GAPDH. Data represent the means ± SD of 3–4 experiments. ^a^P < 0.05 compared with PMA-treated SCi cells.

In an attempt to investigate the involvement of NRF2 in macrophage differentiation, we compared phenotypic changes of *NRF2*-silenced U937 with that of the control cells, following PMA treatment. The NRF2 shRNA-expressing stable cell line (NRF2i) and nonspecific shRNA-expressing control U937 (SCi) were established in our previous study [29]. It was confirmed that stable NRF2i U937 cells showed a 63% reduction in *NRF2* mRNA level when compared to SCi U937 control cells (Fig 1A). Accordantly, NQO1 and AKR1c1 levels were repressed by 55% and 37% of those of the SCi controls, respectively (Fig 1B). Cell adhesion is a typical phenotypic change of differentiated macrophages. It was observed that both cell lines adhered following PMA exposure. For this experiment, PMA was solubilized in ethanol as dimethyl sulfoxide (DMSO) has a stimulation effect on monocytes [34]. Of note, *NRF2*-silenced U937 cells showed facilitated adherence: adherent cell number was significantly greater than in the control SCi cells (Fig 1C and 1D). In addition, macrophage-like morphologic changes were notable in the NRF2i group (Fig 1C). These results indicate that PMA-stimulated phenotypic change can be accelerated by NRF2 inhibition.

PMA treatment is known to enhance macrophage surface markers such as CD44 and CD14 [35, 36]. The transcript levels of CD44 and CD14 were increased by PMA treatment in both cell lines; however, the fold increase was substantially higher in *NRF2*-silenced U937 cells (Fig 1E and 1F). Similarly, PMA-inducible level of intercellular adhesion molecule-1 (MCP-1) and fibronectin-1 (FN-1) was higher in *NRF2*-silenced U937 (Fig 1G and 1H). These results suggest that PMA-inducible expression of macrophage-associated genes was facilitated by *NRF2* silencing.

### Expression of PMA-inducible pro-inflammatory cytokines is significantly enhanced in *NRF2*-silenced U937 cells

**Fig 2:**
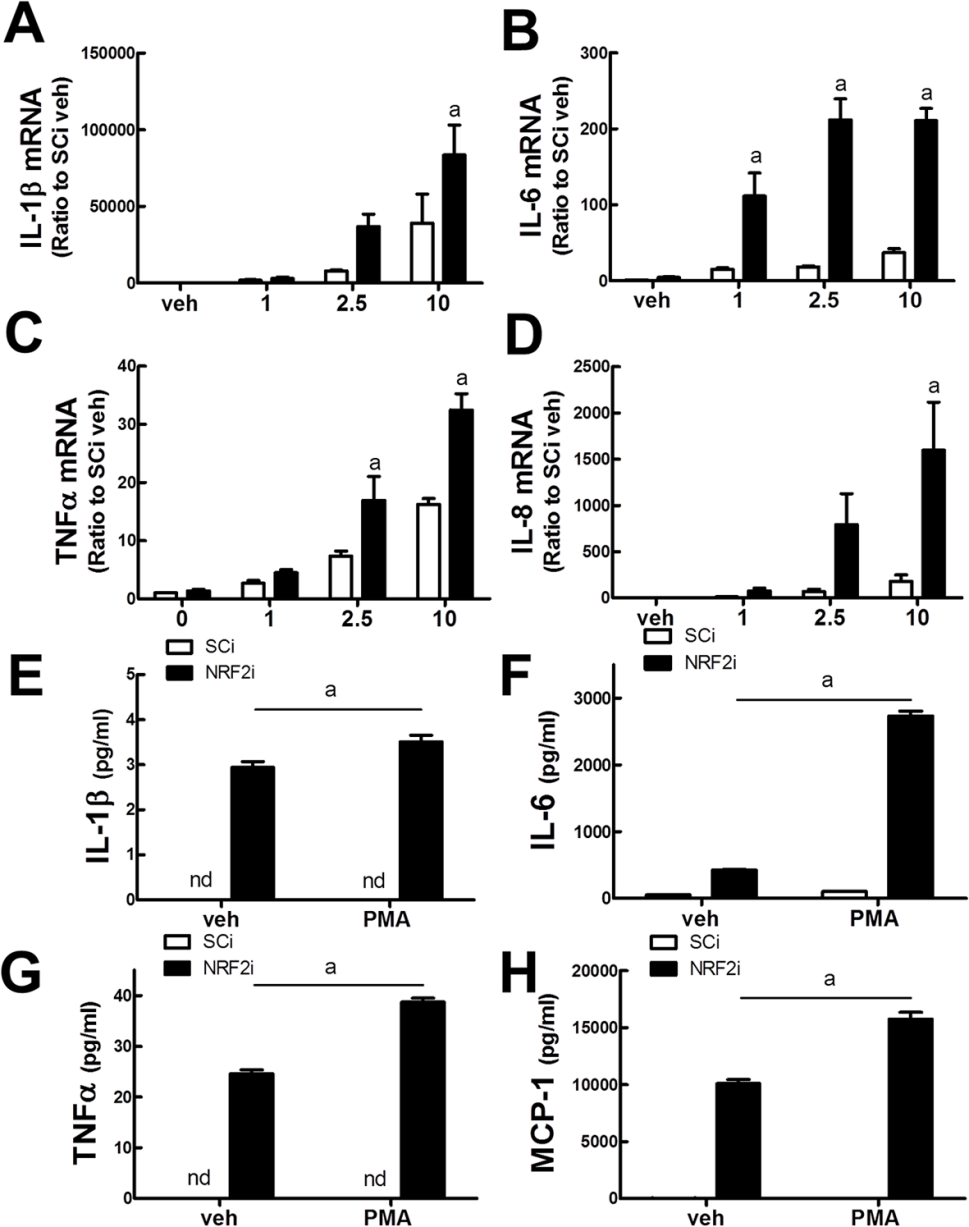
Levels of pro-inflammatory cytokines and chemokine in PMA-treated NRF2i U937 cells. (A-D) SCi and NRF2i U937 cells were incubated with vehicle (ethanol) or PMA (1, 2.5, and 10 ng/mL) for 24 h, and transcript levels of pro-inflammatory cytokines IL-1β (A), IL-6 (B), and TNFα (C), and chemokine IL-8 (D) were determined by real-time RT-PCR analysis. Expression levels of each gene were normalized with respect to the housekeeping gene HPRT or GAPDH. Data represent the means ± SD of 3–4 experiments. ^a^P < 0.05 compared with PMA-treated SCi cells. (E-H) Levels of soluble pro-inflammatory cytokines in PMA-treated NRF2i U937 cells. SCi and NRF2i U937 cells were incubated with vehicle (ethanol) or PMA (10 ng/mL) for 24 h, and IL-1β (E), IL-6 (F), TNFα (G), and MCP-1 (H) levels were monitored in the culture media using the Bio-Plex cytokine assay system. Data represent the means ± SD of 5–6 experiments. ^a^P < 0.05 compared with PMA-treated SCi cells.

Increased expression of cytokines and chemokines is one of the phenotypic characteristics of differentiated macrophages. With the addition of PMA, pro-inflammatory cytokine IL-1β, IL-6, TNFα, and chemokine IL-8 transcript levels were enhanced dose-dependently. Notably, these levels were significantly higher in the *NRF2*-silenced cells than in the control SCi cells (Fig 2A–2D). Next, to confirm that NRF2i cells expressed higher levels of pro-inflammatory cytokines, IL-1β, IL-6, and TNFα protein levels were assessed using the Bio-Plex cytokine assay system. The obtained results consistently indicated that the PMA-inducible production of these cytokines was significantly higher in NRF2i cells (Fig 2E–2G). Additionally, the protein level of MCP-1, a macrophage-secreting chemokine recruiting monocytes, was also significantly higher in NRF2i than in SCi cells (Fig 2H). These results show that *NRF2* gene silencing could enhance the PMA-stimulated differentiation of monocytic U937 cells into pro-inflammatory macrophages.

### *NRF2*-silenced U937 cells retain high ROS levels

**Fig 3:**
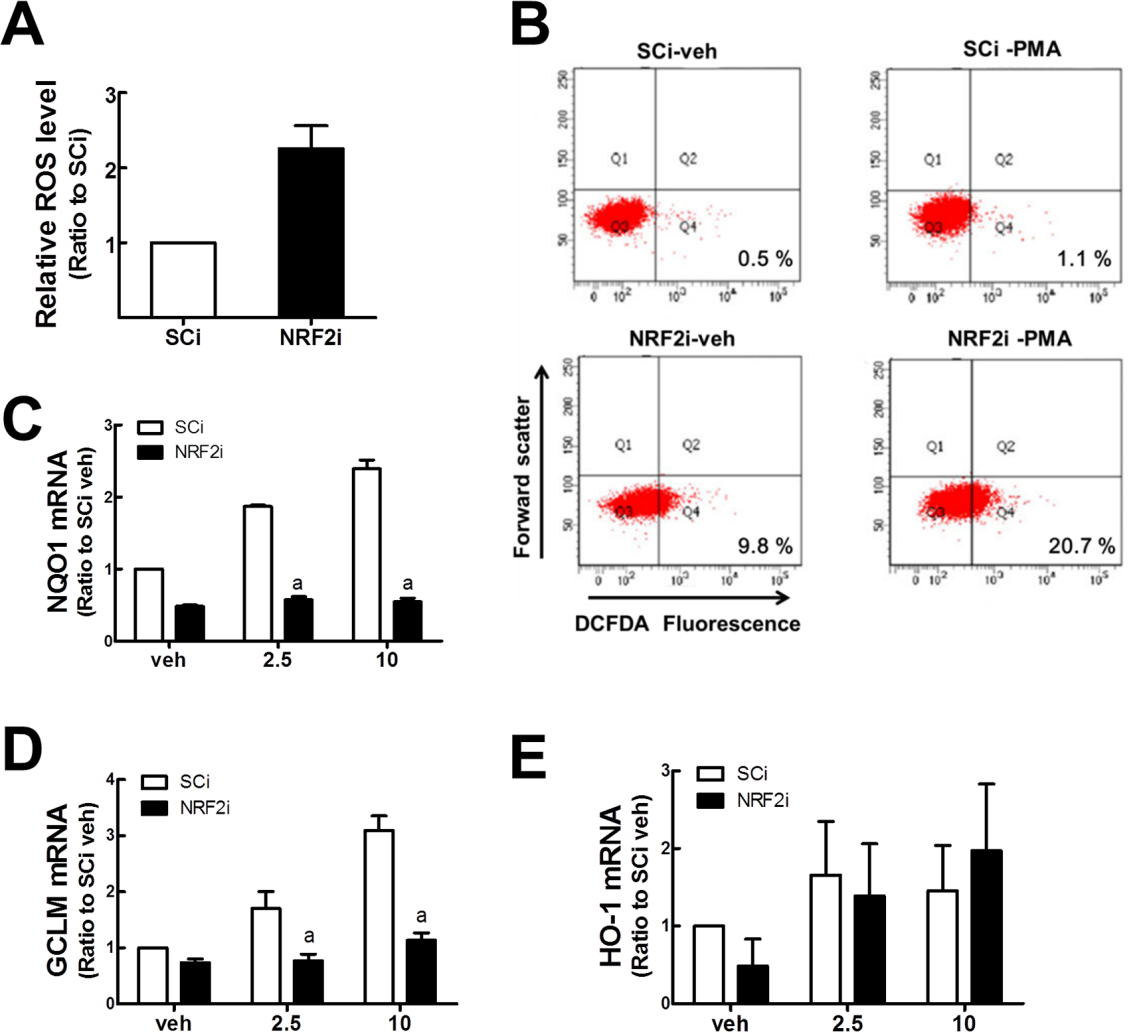
Levels of ROS in NRF2i U937 cells. (A) Cellular level of ROS was monitored in SCi and NRF2i cells. Carboxy-H_2_DCFDA was incubated with cells and its fluorescent intensity was quantified using a fluorocytometry analysis. (B) ROS levels were determined in PMA-treated U937 with a fluorocytometry. PMA (10 ng/ml) was incubated for 24 h and cellular ROS were monitored following DCFDA incubation. (C-E) Transcript levels for NQO1 (C), GCLM (D), and HO-1 (E) were quantified in SCi and NRF2i U937 cells following PMA (2.5 and 10 ng/mL) incubation for 24 h. Data represent the means ± SD of 3–4 experiments. ^a^P < 0.05 compared with PMA-treated SCi cells.

ROS act as important signaling mediators in macrophage differentiation [3]. Due to the reduced expression of its target antioxidant genes, NRF2-inhibited cells may have a higher level of ROS; thus, affecting the differentiation process. Indeed, the NRF2i U937 cells showed higher levels of ROS: the fluorescent intensity from carboxy-H_2_DCFDA-incubated NRF2i cells was 2.25-fold higher than that of SCi cells (Fig 3A). Furthermore, we observed that PMA-induced ROS level is higher in *NRF2*-silenced group: cell populations with DCFDA-derived fluorescence were 1.1% and 20.7% in PMA-treated SCi and NRF2i, respectively (Fig 3B). As for the antioxidant capacity, PMA treatment elevated the expression of NRF2-target genes, such as NQO1 and GCLM in the control U937 cells; however, these increases were largely abolished in the NRF2i U937 cells (Fig 3C and 3D). This indicates that in control cells, PMA-stimulated NRF2-target genes can participate in ROS elimination, whereas the *NRF2*-silenced U937 cells lack this defense system, leading to a further increase in ROS upon PMA treatment. However, HO-1 gene expression did not show a significant difference between the two cell lines (Fig 3E).

### ER stress, cellular Ca^2+^, and PKCα levels are enhanced in *NRF2*-silenced U937 cells

**Fig 4:**
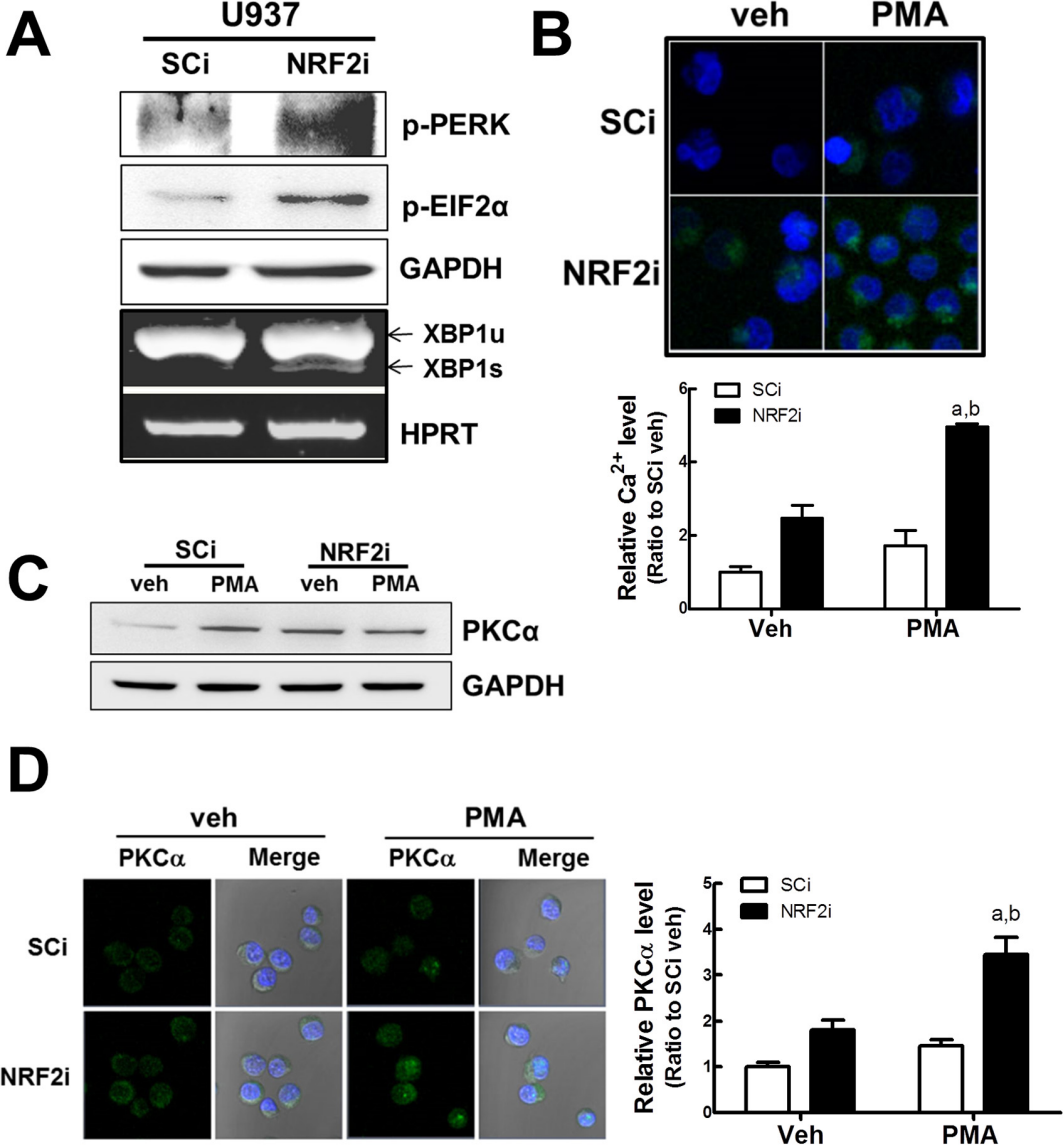
Levels of cellular Ca^2+^ and PKCα in NRF2i U937 cells. (A) Levels of ER stress markers in SCi and NRF2i U937 cells. Protein levels of phosphorylated PERK and EIF2α were assessed using a western blot analysis. Levels for un-spliced and spliced XBP-1 mRNA were quantified in SCi and NRF2i using RT-PCR analysis. (B) Cellular level of Ca^2+^ was monitored in SCi and NRF2i U937 cells. Cells were incubated with Fluo-4 AM (2 μM) for 30 min. Five min after the addition of vehicle (ethanol) or PMA (10 ng/mL), green fluorescence from Ca^2+^-reacting dye was monitored. The fluorescent intensity was quantified using the ZEN software. A confocal microscopic observation was performed with 400× magnification following 4',6-diamidino-2-phenylindole (DAPI) nuclear staining. Data represent the means ± SD of 3–4 experiments. ^a^P < 0.05 compared with the vehicle control of each cell line. ^b^P < 0.05 compared with PMA-treated SCi cells. (C) Western blot analysis of PKCα was performed in SCi and NRF2i U937 cells following PMA (10 ng/mL) incubation for 30 min. Similar blots were obtained in three independent experiments. (D) Immunocytochemical analysis of PKCα. SCi and NRF2i cells were incubated with vehicle (ethanol) or PMA (10 ng/mL) for 24 h, and PKCα cellular level was assessed following antibody incubation. A confocal microscopic observation was performed with 400× magnification following DAPI nuclear staining.

To link increased cellular ROS to PMA-enhanced differentiation, we next speculated that NRF2i cells might have differential levels of cellular Ca^2+^ due to disturbed ER homeostasis. When ER stress markers were monitored, level of phosphorylated PERK protein was higher in *NRF2-*silenced U937 cells than in control cells (Fig 4A). PERK is one of ER stress sensing molecules and its activation suppresses protein synthesis through phosphorylation of the translation initiation factor EIF2α. In parallel with increased p-PERK, p-EIF2α level was higher in *NRF2*-silenced U937. As another ER stress maker, levels of spliced XBP1 reflect IRE1α activity. In addition to activated PERK signaling, we observed that NRF2i U937 retained spliced XBP1 mRNA. These results imply that *NRF2*-silenced U937 is in a state of ER homeostasis disturbance presumably due to high ROS level. As a result, when cellular Ca^2+^ level was monitored with Fluo-4 staining it was observed that Ca^2+^ level was 2.4-fold higher in NRF2i than in control SCi cells (Fig 4B). Ca^2+^ is a strong activator of PKC. When PKCα protein level was assessed by western blot, the basal level of PKCα was higher in NRF2i U937 than in the control (Fig 4C). An immunocytochemical analysis of PKCα confirmed this difference: the fluorescent intensity from the reacted PKCα antibody was relatively higher than in the NRF2i cells (Fig 4D). Notably, in addition to the basal level, PMA-inducible levels of Ca^2+^ and PKCα were different in both cell lines. PMA-mediated Ca^2+^ increase (Fig 4B) and PKCα fluorescent intensity (Fig 4D) were significantly higher in NRF2-silenced U937 than that in SCi cells, although western blot analysis did not show any difference. All together, these results indicate that high ROS causes an enhanced response of NRF2i cells to PMA by activating Ca^2+^ and PKCα signaling.

### NFκB signaling is involved in PMA-stimulated NRF2i cell differentiation

**Fig 5:**
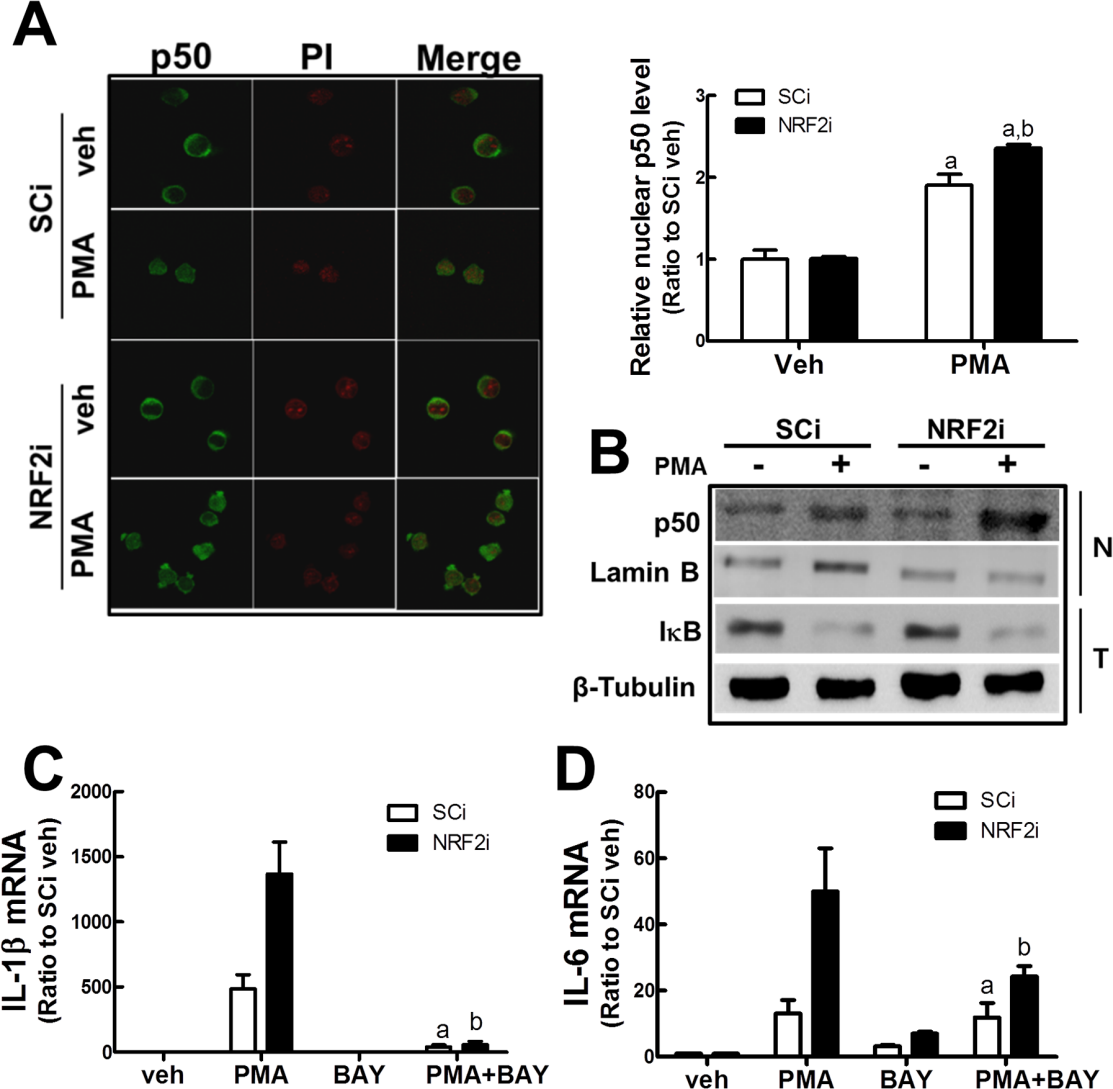
Effect of NFκB inhibition on PMA-stimulated NRF2i cell differentiation. (A) Immunocytochemical analysis of NFκB p50. SCi and NRF2i U937 cells were incubated with vehicle (ethanol) or PMA for 6 h, and levels of p50 were determined using confocal microscopic observation (400× magnification). Nuclear staining was performed following PI incubation. The bar graph represents p50 nuclear levels. (B) Protein levels of p50 and IκB were determined by western blot. (C-D) The cells were incubated with the NFκB inhibitor BAY11-4082 (BAY, 10 nM) or BAY+PMA for 24 h. Transcript levels for IL-1β (C) and IL-6 (D) were determined using RT-PCR analysis. Data represent the means ± SD of 3 experiments. ^a^P < 0.05 compared with PMA-treated SCi. ^b^P < 0.05 compared with PMA-treated NRF2i.

NFκB is the primary transcription factor regulating the expression of pro-inflammatory cytokines [14]. When cellular NFκB p50 protein level was monitored using an immunocytochemical analysis, the basal p50 was localized in the cytoplasm in both cell lines: whereas, the localization shifted to the nucleus following PMA incubation (Fig 5A). Notably, the level of PMA-inducible nuclear p50 accumulation was greater in *NRF2*-silenced U937 cells. An immunoblot analysis also confirmed that nuclear NFκB p50 level is higher in PMA-treated NRF2i cells; although, a similar level of IκB decrease was observed in both cell lines (Fig 5B). These results show that PMA-stimulated NFκB signaling activation is more profound in the NRF2i cells, and this involvement was evidenced by a pharmacological inhibitor treatment. When BAY11-7082 (10 μM), an inhibitor of IκB kinase, was incubated in cells for 24 h, the increase in IL-1β and IL-6 was largely abolished in NRF2i U937 (Fig 5C and 5D).

### Inhibition of ERK signaling abolishes PMA-stimulated NRF2i cell differentiation

**Fig 6:**
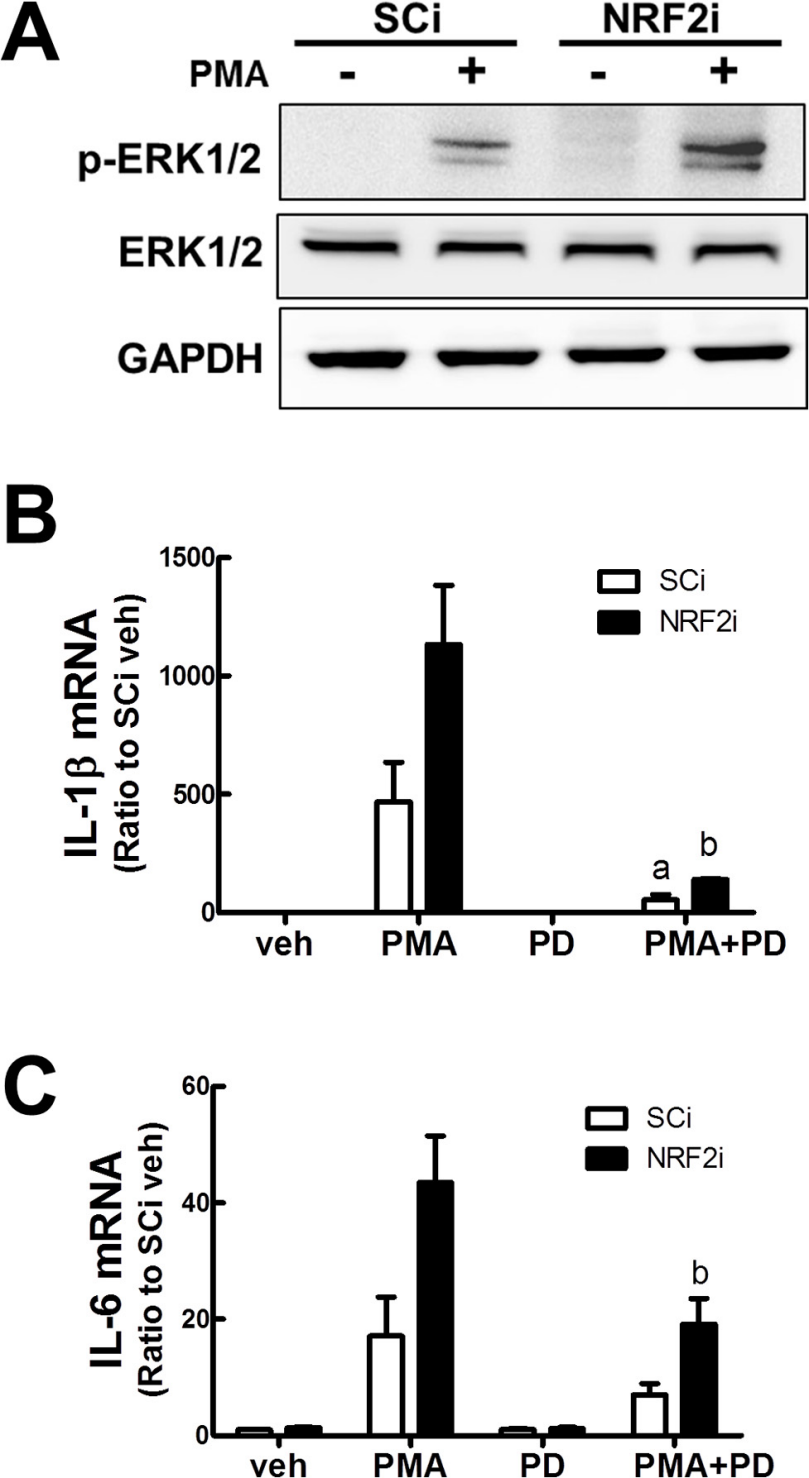
Effect of ERK inhibition on PMA-stimulated NRF2i differentiation. (A) Protein levels of phosphorylated ERK1/2 and total ERK1/2 were determined by western blot. SCi and NRF2i cells were incubated with PMA (10 ng/mL) for 30 min. (B-C) ERK1/2 inhibitor PD98059 (PD, 30 nM) was added to the cells for 24 h. Transcript levels for IL-1β (B) and IL-6 (C) were determined using RT-PCR analysis. Data represent the means ± SD of 3 experiments. ^a^P < 0.05 compared with PMA-treated SCi. ^b^P < 0.05 compared with PMA-treated NRF2i

PKC-mediated ERK stimulation is known to activate NFκB to induce cytokine expression [16, 17]. In an attempt to elucidate the involvement of ERK signaling in NRF2i differentiation, phosphorylated ERK level was assessed following PMA treatment. Level of phosphorylated ERK1/2 was elevated in NRF2i cells, implying the activation of PMA-stimulated ERK signaling mediates cytokine production (Fig 6A). In addition, the incubation of NRF2i with ERK inhibitor PD98059 (30 μM) largely blocked PMA-inducible IL-1β and IL-6 transcription (Fig 6B and 6C). These data indicate that PKCα-ERK-NFκB signaling mediates PMA-stimulated NRF2i cell differentiation.

## Discussion

**Fig 7:**
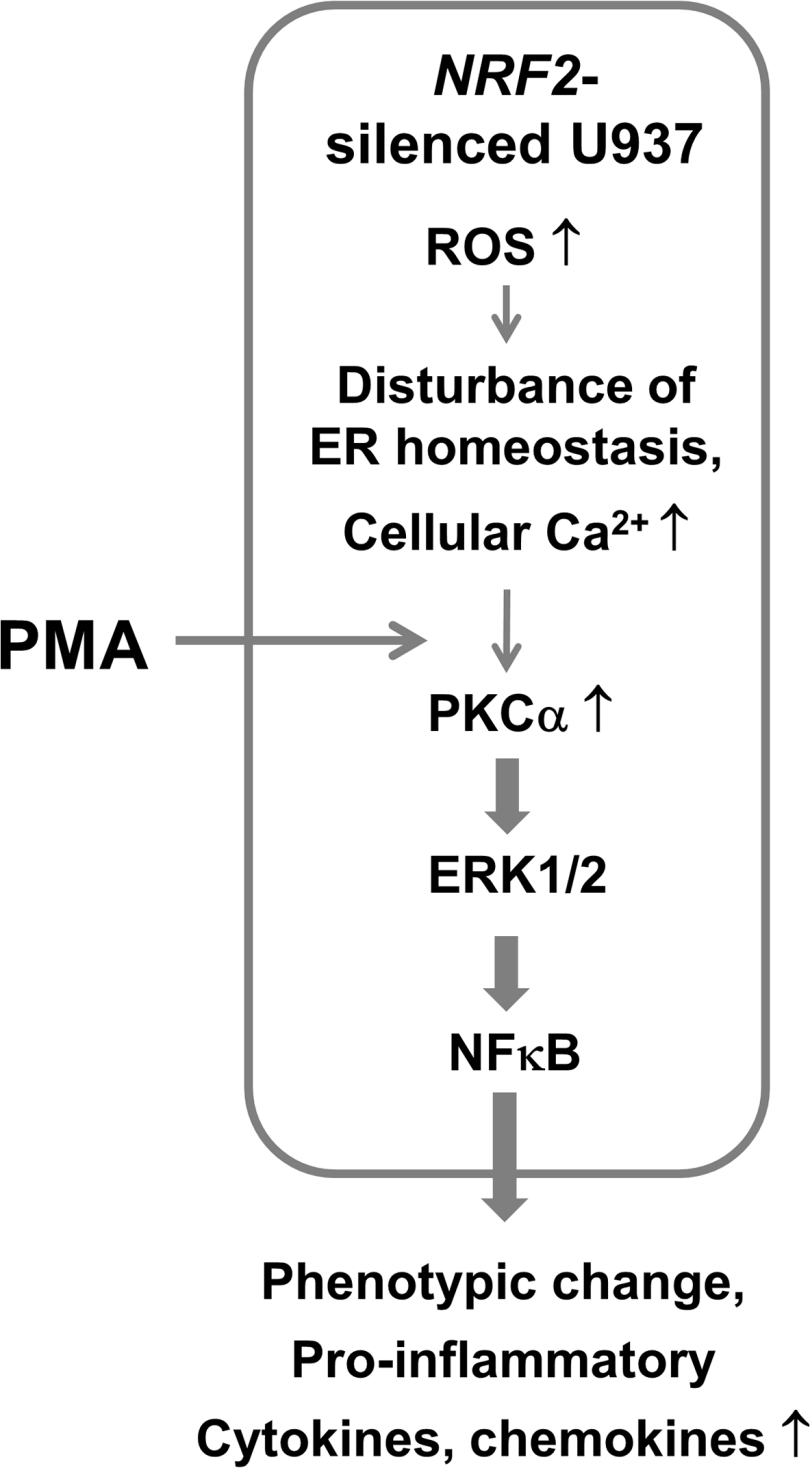
A hypothetical model of pro-inflammatory macrophage differentiation of NRF2i U937 cells. Low level of NRF2 facilitates U937 differentiation into pro-inflammatory macrophages. The *NRF2*-silenced U937 cells retain increased ROS and ER homeostatic disturbance, which are presumably resulting in elevated cellular Ca^2+^ and PKCα levels. Hence the PMA-stimulated PKCα activation is amplified in these knockdown cells, leading to accelerated macrophage differentiation via ERK1/2-NFκB signaling pathway.

In immunogenic conditions, circulating monocytes rapidly differentiate into macrophages. Classically activated macrophages produce a wide array of inflammatory cytokines and chemokines, leading to the recruitment of inflammatory cells at the site of infection and the assistance of T cells, which serve as pro-inflammatory macrophages [1, 3, 37]. Stimulators for classically activated macrophages include the microbial component lipopolysaccharide (LPS), interferon-γ (IFNγ), IL-1, and TNFα. These stimuli activate downstream signaling such as NFκB to transactivate the expression of inflammatory genes [5]. Alternatively activated macrophages have been found in conditions with IL-4 receptor activation, and these macrophages produce immunosuppressive cytokine IL-10 and multiple growth factors, including fibroblast growth factor [38]. Overall, these macrophages, unlike classically activated pro-inflammatory macrophages, promote tissue healing by resolving inflammatory response. ROS, which are produced during macrophage activation, are an important a signaling component toward inflammatory responses in macrophages [3, 5]. Nonetheless, the exact molecular mechanism on how the ROS-modulating system is associated with macrophages differentiation and activation remains unclear. In the current study, we investigated the role of NRF2 in monocyte-to-macrophage differentiation using the PMA-stimulated U937 model. The *NRF2*-silenced U937 cells demonstrated increased ROS and ER homeostasis disturbance, which are presumably causing elevated intracellular Ca^2+^ and PKCα levels. Furthermore, the expression of pro-inflammatory cytokines was higher in *NRF2*-silenced U937, indicating that U937 cell differentiation into pro-inflammatory macrophages is facilitated by *NRF2* knockdown (the hypothetical model is shown in Fig 7).

NRF2 involvement in macrophage function has been demonstrated in multiple studies. In a study by Ishii et al., Nrf2 was found to be a critical factor in HO-1 expression in mouse peritoneal macrophages [39]. A study by Liu et al. showed that the induction potency of Nrf2 target genes is closely related with anti-inflammatory effects in mouse peritoneal macrophages, which implies a protective role of Nrf2 in macrophages inflammation [40]. Macrophages from *nrf2*-null mice showed enhanced TNFα production and NFκB activity following LPS challenge [28]. Similarly, in peritoneal foam cell macrophages from *nrf2*-null mice, LPS-stimulated pro-inflammatory response was aggravated when compared to that in wild-type macrophages [27]. As *in vivo* evidence, in a model of ovalbumin-induced asthma, *nrf2* deficiency enhanced airway inflammation and asthma [41]. When the bone marrow from *nrf2*-null mice was transplanted in low-density lipoprotein (LDL) receptor-knockout mice, *nrf2* deletion promoted pro-inflammatory activation of macrophages and enhanced foam cell formation, leading to aggravated atherosclerosis [42]. All together, these reports strongly support the inhibitory role of NRF2 in macrophage-mediated inflammatory response. As an underlying mechanism, a study by Kong et al. have shown that *nrf2*-null macrophages retained a high level of ROS through NADPH oxidase activation, which resulted in Toll-like receptor-4 (TLR4) signaling amplification and greater mortality by sepsis shock [43]. Together with these reports, our results support the anti-inflammatory role of NRF2 by showing the involvement of NRF2 in macrophage differentiation.

Several lines of evidence suggest that NRF2 may play a specific role in cell differentiation. Stable overexpression of Nrf2 in mouse osteoblasts interferes with Runt-related transcription factor 2 (Runx2)-dependent gene expression for osteoblast differentiation [44]. Similarly, osteoblast number in bone is high in *nrf2*-knocout mice, suggesting the negative role of Nrf2 in osteoblast differentiation [45]. In addition to osteoblast differentiation, *nrf2* deficiency promotes the NFκB ligand (RANKL)-stimulated osteoclast differentiation [46]. Similarly, *nrf2* overexpression significantly represses the levels of chondrocyte differentiation markers in mouse [47]. These findings support the negative role of NRF2 in cell differentiation; however, contrasting results showing the positive role of NRF2 have been reported. Isoliquiritigenin facilitates monocytic differentiation of HL-60 cells by up-regulating NRF2 [48]. Nrf2 promotes the differentiation of the mouse neuroblastoma cells, and primary neurons from *nrf2*-null mice show a more delayed differentiation than do wild-type neurons [49]. Although the effects of NRF2 are contrasting depending on the cell type, all of these reports indicate that NRF2-mediated redox modulation plays an important role in the process of cell differentiation. In line with these findings, our results demonstrate that NRF2 may negatively regulate monocyte’s differentiation into pro-inflammatory macrophages using the PMA treated U937 model. It is noteworthy that the cell lines such as U937 and THP-1 has the limitations to be used as pro-monocytic or monocytic cells. Even though U937 and THP-1 cells exhibit the macrophage-similar phenotypic change, which is accompanied by transcriptional alterations, following the exposure to cytokines, PMA, and vitamin D_3_, there has been variability in their responses depending on the type, duration, and intensity of stimuli [7–9, 35, 50]. Therefore, there is a possibility that the negative link of NRF2 to U937 differentiation is not a generalized phenomenon. Indeed, in an experimental system with vitamin D_3_-induced differentiation of U937, the treatment with NRF2 activator, carnosic acid, accelerated U937 differentiation into monocytes [51]. Nonetheless, it is likely that NRF2 negatively engages in pro-inflammatory macrophage differentiation when considering numerous reports showing the aggravated inflammation in *nrf2* knockout cells and animals [27, 28, 39–42]. This relationship further supports the potential therapeutic application of NRF2 activators to inflammatory diseases by modulating pro-inflammatory macrophage differentiation. Seven chemical classes of NRF2 inducers, including isothiocyanates, represented highly correlated anti-inflammatory potencies in mouse peritoneal macrophages [40]. *Nrf2*-null mice are more sensitive to LPS- or TNFα-stimulated lung inflammation, and these mutant mice show a lower survival in experimental sepsis shock [28]. Sulforaphane treatment inhibited LPS-stimulated production of IL-1β and TNFα in wild-type macrophages, whereas *nrf2*-null macrophages did not show anti-inflammatory effect [52].

ER stress can be caused by the disturbance of ER homeostasis, which can be resulted from various signals, including accumulation of misfolded/oxidized proteins and inhibition of protein glycosylation [21, 53, 54]. Particularly, excess ROS are a strong stimulator for ER stress. Since ER protein folding is highly sensitive to ROS, elevated cellular ROS interfere with the process of protein folding and further oxidize ER proteins. Consequently, disturbed ER homeostasis activates ER stress signaling pathway. At the same time, the change in Ca^2+^ homeostasis is notable: ER-based Ca^2+^ channel is known to be affected by ER stress and consequently cytosolic Ca^2+^ level increases [53, 54]. In our *NRF2*-silenced U937 cells, due to repressed expression of NRF2 target genes, such as GSH generating system, ROS level was high, and this is likely to be the direct cause of ER stress and Ca^2+^ increase. Accordantly, we observed that the basal level of PKCα was high in *NRF2*-silenced U937 cells. Moreover, PMA-stimulated Ca^2+^ and PKCα levels were higher in these knockdown cells. Eventually these changes lead to facilitated U937 adhesion and enhanced production of pro-inflammatory cytokines. Of note, it is apparent that *NRF2* knockdown amplified PMA signaling to cell differentiation. In normal cells, it is known that ER stress can activate NRF2 signaling via PERK phosphorylation [55], whereas this compensatory ROS-defense mechanism is impaired in *NRF2*-inhibited cells. Additionally, PKC is known to activate NADPH oxidase to increase ROS production [56]. At the same time, there is evidence that PKC directly activates NRF2 signaling via phosphorylation of NRF2 [57]. Indeed, we observed that PMA incubation enhanced NRF2 target NQO1 and GCLM gene expression in SCi but not in NRF2i U937 cells. Therefore, *NRF2*-silenced U937 cells cannot counteract PKC-stimulated ROS increase, and this eventually may amplify the ROS-ER stress-Ca^2+^-PKCα axis to produce pro-inflammatory cytokines.

Collectively, our study provides the evidence that NRF2 plays a negative role in U937 differentiation into pro-inflammatory macrophages following PMA treatment. As an underlying mechanism, we showed that *NRF2*-silenced U937 cells showed an enhanced response to PMA through the activation of PKCα-ERK-NFκB signaling. These results further support the role of NRF2 in macrophages during inflammatory responses and associated diseases.
